# Domain-Specific Evaluation of Exergame Metrics Among Older Adults With Mild Neurocognitive Disorder: Secondary Analysis of 2 Randomized Controlled Trials

**DOI:** 10.2196/65878

**Published:** 2025-05-21

**Authors:** Wanda Kaiser, Eling D de Bruin, Patrick Manser

**Affiliations:** 1 Department of Neurobiology, Care Sciences and Society Karolinska Institutet Huddinge (Stockholm) Sweden; 2 Department of Health OST - Eastern Swiss University of Applied Sciences St.Gallen Switzerland; 3 Department of Health Sciences and Technology ETH Zurich Zurich Switzerland

**Keywords:** exergaming, cognition, neurocognitive disorder, game metrics, older adults, performance progression

## Abstract

**Background:**

Exergame-based training enhances physical and cognitive performance in older adults, including those with mild neurocognitive disorder (mNCD). In-game metrics generated from user interactions with exergames enable individualized adjustments. However, there is a need to systematically investigate how well such game metrics capture true cognitive and motor-cognitive performance to provide a more robust basis for personalized training.

**Objective:**

The primary objective was to identify valid game metrics as indicators for in-game domain-specific cognitive performance during exergaming in individuals with mNCD. We also aimed to explore game metric performance changes over time during exergame-based training.

**Methods:**

Data were analyzed from individuals with mNCD who completed a 12-week home-based, exergame-based intervention following the Brain-IT training concept. A cross-sectional analysis was conducted by correlating game metrics with standardized neurocognitive reference assessments. To confirm the alternative hypothesis, we predetermined the following criteria: (1) statistically significant correlation (*P*≤.05; uncorrected; 1-sided) with (2) a correlation coefficient (Pearson *r* or Spearman ρ) of ≥0.4. Visual and curve-fitting longitudinal analyses were conducted to explore game performance changes over time.

**Results:**

Data were available from 31 participants (mean age 76.4, SD 7.5 y; n=9, 29% female). In total, 33% (6/18) of the game metrics were identified as valid indicators for in-game cognitive performance during exergaming. In the neurocognitive domain of learning and memory, these metrics included the mean reaction time (ρ=–0.747), the number of collected items (ρ=0.691), and the precision score (*r*=–0.607) for the game Shopping Tour (*P*<.001 in all cases), as well as the point rate (*P*=.008; *r*=0.471) for the game Simon. In addition, point rate was a valid indicator for executive function (*P*=.006; *r*=0.455) and visuospatial skills (*P*=.02; *r*=0.474) for the games Targets and Gears, respectively. The exploratory longitudinal analysis revealed high interindividual variability, with a general trend of the expected typical curvilinear curves of rapid initial improvements followed by a plateau in performance.

**Conclusions:**

This study demonstrated that metrics reflecting the precision of responses generally performed better than metrics reflecting the speed of responses. These observations highlight the importance of selecting valid game metrics for implementation in exergame designs. Further research is needed to explore the potential of game metrics and identify factors contributing to individual variability in in-game performance and performance progression, as well as identifying and adopting strategies that facilitate individual learning success and thus promote effectiveness in improving health outcomes.

## Introduction

### Background

There is growing interest in interventions that aim to mitigate the physical and cognitive decline associated with aging in older adults [1]. Applying gamified digital health technologies with real-time user behavior and gameplay interactions such as computerized cognitive training games or exergame technology is becoming increasingly popular to address the challenges of physical and cognitive decline in the aging population [2-7]. Within this field, considerable attention has been directed toward exergame-based physical and motor-cognitive training, which is considered more promising than conventional physical, cognitive, or motor-cognitive training to improve cognitive and physical performance in diverse user populations [8-10]. Exergames are defined as “technology-driven physical activities, such as video game play, that require participants to be physically active or exercise in order to play the game” [11]. Exergame-based training typically focuses on incorporating cognitive tasks into motor tasks than can be designed to specifically train a variety of physical (eg, cardiorespiratory, motor, balance, strength, or multicomponent exercises) and cognitive (eg, learning and memory, complex attention, executive function, and visuospatial skills) domains [5]. The positive impact of exergaming on physical and cognitive functions has been observed in healthy older adults [5,8,12-14], as well as in older adults with cognitive impairments such as mild neurocognitive disorder (mNCD) or more severe forms such as dementia [5,15-17].

mNCD describes a critical stage of cognitive decline that goes beyond normal aging without affecting the capacity for independence in everyday activities [18]. Early interventions may effectively influence the course of cognitive decline, making those with mNCD an optimal target population for secondary prevention [19-22]. Several pharmacological and nonpharmacological methods have been proposed to slow down the progression of symptoms accompanying mNCD and improve overall quality of life [20,23]. According to current research, the most effective type of exercise to achieve this goal is to implement physical training, ideally with integrated cognitive tasks (simultaneous motor-cognitive training) [21,24-30]. This type of training has been proposed to operate through various disease-modifying mechanisms, including multisystem effects that reduce mNCD-related neuropathological damage [31,32] and promote synaptogenesis, neurogenesis, and angiogenesis [33,34]. It also helps maintain or increase cognitive reserve [26,35,36], empowering individuals to retain independence in everyday functioning despite neurodegenerative changes [33,34].

Physical and motor-cognitive training may represent the predominant nonpharmacological intervention that effectively mitigates cognitive decline in mNCD [27,30,37,38] and is recommended for secondary prevention of mNCD by a collaborative international guideline [39] as well as a global consensus on optimal exercise recommendations for enhancing healthy longevity in older adults [22]. In this context, exergames provide a promising solution to deliver this type of training [30]. Recent systematic reviews support the use of exergame-based training for improving both physical and cognitive performance in individuals with mNCD. However, studies in this field exhibit high heterogeneity, which weakens the strength of the evidence [15,16,40-42]. Furthermore, recent research suggests that, while exergame-based training may indeed improve cognitive performance, its superiority over traditional training remains uncertain, and further research should investigate the conditions in which exergames are most effective [12].

Several exergame systems have been investigated for older adults and have been shown to effectively promote engagement and motivation [43-46], which largely explains the superior adherence to exergame- or technology-enhanced training compared to adherence to conventional approaches of physical and motor-cognitive activities, exercise, or training [46,47]. Furthermore, exergames offer high value due to the technical adaptability of various elements. However, while many exergames are technically well designed for attractiveness and entertainment, they often lack systematic user adaptability and specificity [48]. Therefore, future exergames should be better tailored to specific target groups as well as individual goals and abilities [5,8,48,49].

To enhance the user adaptability of exergames, it has been recommended to implement real-time adaptive exergame mechanisms that support the dual-flow concept proposed by Sinclair et al [50]. According to this concept, an optimal exergame experience requires a balance between the challenge of the game and the player’s skills, as well as between the intensity of the game and the player’s fitness. A common method for implementing adaptive exergame mechanics is to use the data that are directly collected during exergaming [49]. These data are commonly referred to as in-game metrics or performance measures and capture the real-time interaction between the user and the serious game. Game metrics are quantitative measures that can range from simple measures such as point scores or reaction time to more complex measures that combine various parameters [51]. Game metrics have the potential to improve personalization and individualized progression of training according to users’ performance, provide (real-time) feedback, and monitor gameplay [51-53]. However, there are currently no standards or guidelines for what data should be collected and for what specific purpose [52,54].

By investigating game metrics and understanding how well they can capture true cognitive and motor-cognitive performance, we can provide a robust basis for in-game adjustments and monitoring of performance progression [54,55]. While many studies emphasize the importance of individually tailored training and commonly use in-game metrics for this purpose, only a limited number have analyzed the psychometric properties of in-game metrics. Previous studies that have investigated game metrics from exergames for older adults with and without physical or cognitive impairments have found moderate to strong correlations between some game metrics and clinical assessments [54,56,57]. This suggests that certain game metrics may be useful for measuring physical and cognitive performance. Furthermore, one study demonstrated that game metrics can distinguish between healthy older adults and those with mNCD [56]. In addition, another study explored a difference in exergame performance progression between older adults with and without cognitive impairment [57]. However, further studies are needed with domain-specific evaluation, larger participant samples, and inclusion of participants with a clinical diagnosis of mNCD, as stated by Guimarães et al [57].

### Objectives

The primary objective was to identify valid game metrics as indicators for in-game domain-specific cognitive performance during exergaming in older adults with mNCD. We hypothesized statistically significant correlations between specific game metrics and reference assessments within their corresponding neurocognitive subdomains. As a secondary objective, we aimed to explore game metric performance change over time during a 12-week exergame-based training intervention for older adults with mNCD.

## Methods

### Overview

This study is a secondary analysis of data from 2 randomized controlled trials (RCTs) that aimed to evaluate the feasibility (feasibility study [FS]; NCT04996654) [58] and effectiveness (effectiveness study [ES]; NCT0538707) [59] of an individually tailored home-based exergame-based training concept specifically for the secondary prevention of mNCD developed within the Brain-IT project [60]. The training concept is rooted in years of iterative and user-centered co-design, development, testing, and evaluation with continuous patient and public involvement [61]. It serves as a guideline for the implementation of the training by providing algorithmic decision trees for the structure, content, and individualized tailoring of the training and can be implemented using different commercially available hardware [59].

The project was structured in 3 phases. In phase 1, we combined a comprehensive literature synthesis [61] with qualitative research, including primary end users (individuals with mNCD), secondary end users (multidisciplinary health and care professionals), other exergaming researchers, and experts from the exergaming industry [62], to elaborate a set of design requirements for the Brain-IT training concept. In phase 2, possible concepts were co-designed and developed based on the requirements defined in phase 1. The first prototype of the Brain-IT training concept [59,61] then entered the iterative cycle of feasibility, usability, safety, and acceptance testing and integrating of findings for further development until an “acceptable” solution was achieved (refer to the FS [58], the data from which are analyzed in this study). This process resulted in a novel intervention type specifically targeting various relevant mechanisms of action to alleviate the pathological state of mNCD by combining, for the first time, exergame-based motor-cognitive training with biofeedback-guided resonance breathing as an adjunct neuromodulatory intervention [59]. In phase 3, we confirmed the effectiveness (refer to the ES [59], the data from which are analyzed in this study) of the Brain-IT training in improving global cognitive performance as well as immediate and delayed verbal recall. We observed that the training not only could effectively slow down cognitive decline in comparison to usual care but also resulted in 55% of participants showing clinically relevant improvements in cognitive performance [59].

We compiled this manuscript according to the latest version of the CONSORT (Consolidated Standards of Reporting Trials) Statement for Randomized Trials of Nonpharmacologic Treatments (Tables S1 in Multimedia Appendix 1). As this is a secondary analysis of 2 RCTs, not all original trial design elements may be fully applicable or reported in this paper as they were not relevant for the specific analysis conducted (for full reproducibility, please also refer to the published FS [58], the study protocol for the ES [63], the ES itself [59], and the training concept—Supplementary File 2 of the ES [59]).

### Ethical Considerations

All original study procedures were carried out in accordance with the Declaration of Helsinki. The original study protocols were approved by the ETH Zurich Ethics Commission (EK 2021-N-79) for the FS and the Ethics Committees of Zurich and Eastern Switzerland (EK-2022-00386) for the ES.

All study participants were fully informed about the study procedures in person (at the interested person’s home or at one of the study centers depending on their preference) through verbal explanations and an information sheet. After sufficient time for consideration (ie, at least 24 hours after handing out the study information sheet [which was approved by the corresponding ethics committee] but, on average, approximately 1 week), suitable patients willing to participate in the study provided written informed consent in a second in-person meeting with one of the trained investigators of the study team at the home of the interested person or at one of the study centers. The original informed consent forms allowed for the secondary analyses conducted in this study as all study participants provided consent for (1) original datasets being made available in a publicly accessible repository in deidentified form and (2) the transfer of encrypted data for research purposes. Only deidentified data were analyzed for this study (for more detail, see the Data Availability section).

No compensation was granted to participants, but detailed feedback on individual performance as well as the study outcomes in general was provided at the end of the studies.

### Study Design and Participants

Recruitment for the 2 parallel-group and single-blinded (the outcome evaluator of pre- and postintervention measurements was blinded to group allocation) RCTs took place between July 2021 and October 2023 in collaboration with health care facilities in the larger area of Zurich, Switzerland. The eligibility criteria for study participants have been published previously [58,59,63] and are listed in Textbox 1 [64-68].

**Eligibility criteria for participation in the 2 randomized controlled trials, the data from which were analyzed in this study. The eligibility criteria were checked by a trained investigator of the study team. Information about the clinical diagnosis and comorbidities was obtained from the collaborating recruitment partners.**

**Inclusion criteria**

- Clinical diagnosis of mild neurocognitive disorder according to the *International Classification of Diseases, 11th Revision* [64], or the *Diagnostic and Statistical Manual of Mental Disorders, 5th Edition* [65], OR patients screened for mild cognitive impairment (MCI) according to the following criteria: (1) informant (ie, health care professional)-based suspicion of MCI confirmed by (2) an objective screening of MCI based on the validated German version [66] of the Quick Mild Cognitive Impairment screen [67], with a recommended cutoff score for cognitive impairment (or dementia) of <63/100 [68] while not falling below the cutoff score for dementia (ie, <45/100) [68]
- German speaking
- Able to stand for at least 10 minutes without assistance

**Exclusion criteria**

- Mobility impairments (ie, gait and balance) that prevented experiment participation
- Presence of additional, clinically relevant (ie, acute or symptomatic or both) neurological disorders (ie, epilepsy, stroke, multiple sclerosis, Parkinson disease, brain tumors, or traumatic disorders of the nervous system)
- Presence of any other unstable or uncontrolled diseases (eg, uncontrolled high blood pressure and progressing or terminal cancer)

After successful recruitment, participants underwent a premeasurement (see the Reference Assessments subsection in the Outcomes section) at the study center located at ETH Hönggerberg and were randomly assigned in a 1:2 (FS) or 1:1 (ES) ratio to either the control or intervention group. This secondary analysis investigated only the data from participants allocated to the intervention groups who engaged with the exergame-based training.

There were no important changes to the trial design and study setting after commencement that were relevant to the analyses presented in this paper. More details can be found in the published FS [58] and ES [59].

### Training Intervention

Following the premeasurement and group allocation, a study investigator installed the exergame system in the participants’ homes, where the training sessions took place. A standardized familiarization session was conducted to introduce the exergame system (ie, 4 games from the first scheduled training session [at level 1] were introduced and played for 2 minutes each). The exergame system Senso Flex (Dividat AG; hardware: prototype version 2; software: version 22.4.0-360-gf9df00d5b) was used [69]. The participants were instructed to follow the Brain-IT training concept [59] for the 12-week intervention, which consisted of completing an instructed minimum of 5 training sessions per week. Each training session had a duration of 24 minutes and included 6 motor-cognitive games and 3 resonance breathing exercises. Approximately one-third of the sessions were supervised by a study investigator, with more supervision provided at the beginning of the intervention and less toward the end [59]. The intervention was personalized and individually adapted based on several predefined parameters and included games for the neurocognitive domains of learning and memory, executive function, complex attention, and visuospatial skills [59,70]. The relevant parameters for this study were (1) the focus on participants’ main cognitive deficits through an individual constellation of games and (2) the individual adaptation of the standardized difficulty levels (levels 1-10), with each participant starting at level 1 [59].

The parameters for determining the 10 game levels were defined through agreement between an experienced neuropsychologist and the research team and were extensively tested [71]. According to the Brain-IT training concept, level 1 should refer to an introductory level (ie, even most impaired patients with mNCD should be able to play the game at the first trial), and in level 10 (ie, “healthy functioning” level), the game demands are expected to be challenging but doable for an average healthy older adult. The remaining levels are defined to increase neurocognitive demands consecutively and regularly from level to level. In the definition of these 10 game levels, we integrated the methodology by Huber et al [72], who established an extended taxonomy supporting motor-cognitive learning. This methodology provides a framework for structuring training to specifically integrate models of skill acquisition [73,74] that has also been described to apply to cognitive skill learning and relearning [75].

For more details on the specific exergames, as well as all algorithmic decisions for the personalized design and individualized progression of the training, including a description of all games and game level settings, we kindly refer interested readers to the published Brain-IT training concept [59]. To ensure replicability, the Brain-IT training concept was planned and reported according to the Consensus on Exercise Reporting Template [76]. The training concept provides specific instructions on how to adapt the training when implemented with alternative hardware and software solutions (Supplementary File 2 of the ES [59]).

### Outcomes

#### Demographics

During the premeasurement, participants provided self-reported information on sociodemographic variables, including age, sex, BMI, and years of education. The characterization of the etiology of mNCD was derived from the diagnostic information provided by the collaborating recruitment partners. Global cognitive performance was assessed using the validated German version [66] of the Quick Mild Cognitive Impairment screen [77,78].

#### Game Metrics

A total of 14 different exergames were implemented in the Brain-IT training concept. During the development phase of the training concept, neuropsychologists assigned each exergame to its primary targeted neurocognitive function to ensure its content validity [61]. However, it should be mentioned that exergames typically involve and train multiple neurocognitive functions. The exergames were assigned to the primary targeted neurocognitive domains of (1) learning and memory, (2) executive function, (3) complex attention, and (4) visuospatial skills. In addition, they were subcategorized into neurocognitive subdomains [61]. For this study, we analyzed 8 exergames, with 2 games representing each neurocognitive domain, as listed in Table 1. These games were selected based on data availability (ie, games that were frequently played during the intervention and were introduced to most, if not all, participants to ensure robust analyses). We collected all game metrics that were computed by the exergame system and, therefore, available for analyses (ie, no selection or exclusion of specific metrics). A detailed description of the game’s content, parameters to adapt, and game metrics (referred to as “performance measures”) can be found in the Brain-IT training concept [59].

**Table 1:** Overview of the analyzed games and their outcome variables categorized by neurocognitive domains and subdomains^a^.

Neurocognitive domain and subdomain		Exergame		Neuropsychological assessment—outcome variable
		Name	Outcome variable	
**Learning and memory**				
	Free recall	Shopping Tour	Mean reaction time (ms) Number of collected items Number of mistakes Precision score (%)	WMS-IV-LM 1^b^—free recall (total point score)
	Serial recall	Simon (color and number)	Mean reaction time (ms) Point rate	PEBL^c^ DSF^d^ (total point score)
**Executive function**				
	Working memory	Nomis (color and number)	Mean reaction time (ms) Point rate	PEBL DSB^e^ (total point score)
	Planning	Targets	Hits (number per min) Misses (number per min) Point rate	HOTAP (points × min^–1^)
**Complex attention**				
	Selective attention	Habitats	Mean reaction time (ms) Point rate	TAP^f^ Go-No go (median reaction time [ms])
	Processing speed	Simple	Mean reaction time (ms) Point rate	PEBL TMT-A^g^ (completion time [s])
**Visuospatial skills**				
	Visual perception	Gears	Mean reaction time (ms) Point rate	PEBL MRT^h^ (performance score)
	Visuoconstructional reasoning	Tetris	Game score (min–1)	PEBL MRT (performance score)

^a^The categorization of exergames into the neurocognitive domains and subdomains was derived from the published Brain-IT training concept. Accordingly, each game was categorized into the primary neurocognitive domain and subdomain being trained (and secondary subdomain in the case of a game that focuses on more than one neurocognitive domain or subdomain). The categorization was made through agreement between an experienced neuropsychologist and the research team to ensure the content validity of the exergames used to train each neurocognitive domain or subdomain. The variables listed in the Outcome variables column under Exergame describe all game metrics that were computed by the exergame system and, therefore, available for analyses (ie, no selection or exclusion of specific metrics). For each of these games, the most appropriate reference assessment was selected from the available published datasets. Specifically, we used the neurocognitive domains or subdomains that the specific games primarily trained (as defined in Table 1 of the published Brain-IT training concept [59]) as references and selected the reference assessment that measured the same primary neurocognitive domain or subdomain from the available published datasets.

^b^WMS-IV-LM 1: Wechsler Memory Scale–Fourth Edition–Logical Memory subtest part 1.

^c^PEBL: Psychology Experiment Building Language.

^d^DSF: digit span forward.

^e^DSB: digit span backward.

^f^TAP: Test of Attentional Performance.

^g^TMT-A: Trail Making Test part A.

^h^MRT: Mental Rotation Task.

#### Reference Assessments

Data from several domain-specific neuropsychological assessments collected during the premeasurement were used as reference. For each assessment, the participant received standardized instructions from a study investigator, who also ensured that the participant understood the task and the procedure. The assessments and the outcome variables used for this analysis are listed in Table 1. The study protocol of the ES [63] provides detailed information about the assessments. For each of the games, the most appropriate reference assessment was selected from the available published datasets. Specifically, we used the neurocognitive domains or subdomains that the specific games primarily trained (as defined in Table 1 of the published Brain-IT training concept [59]) as references and selected the reference assessment that measured the same primary neurocognitive domain or subdomain from the available published datasets, as listed in Table 1 of the published Brain-IT training concept [59]. A brief description of the assessments and their function is provided in this section.

For the neurocognitive domain of learning and memory, the German version of the Logical Memory subtest part 1 from the Wechsler Memory Scale–Fourth Edition [79,80] was used to measure the neurocognitive subdomain of free recall. Data for the subdomain of serial recall were obtained from the digit span forward test of the Psychology Experiment Building Language (PEBL) [81], a computer-based software platform for psychological experiments. Executive function was assessed using the HOTAP picture-sorting test part A [82] to evaluate planning skills and the PEBL digit span backward test [81] to evaluate working memory. For the neurocognitive domain of complex attention, data from the PEBL Trail Making Test part A [81] for processing speed and data from the Go-No go task 1 of 2 from the Test of Attentional Performance [83,84] for selective attention were used. Finally, we considered data from the computerized PEBL Mental Rotation Task [81] that is based on the classic mental rotation task to assess visuospatial skills.

### Data Analysis and Statistical Methods

#### Overview

Statistical analysis was conducted using R (version 2023.12.0+369; R Foundation for Statistical Computing). Data are reported as mean and SD for interval scales or continuous data and as counts and percentages for categorical variables. Descriptive statistics were calculated for all outcome variables. The assumption of normality distribution was checked through the Shapiro-Wilk test alongside a visual examination of the data [85].

#### Primary Objective: Cross-Sectional Analysis

For the primary objective, we used a cross-sectional analysis. Only the game metrics from the first time that each participant played the first level of difficulty were analyzed. There were 2 main reasons for this. First, not all participants played the same number of repetitions of a game level due to individual adaptations, and second, we aimed to minimize the influence of learning effects in this cross-sectional analysis. This analysis included all participants who played the corresponding game at least once. The training concept underwent minor adjustments between the FS and the ES. One such adjustment involved extending the duration of certain games from 2 to 3 minutes. Metrics that were affected by this change and are time dependent were documented as metrics over time (min^–1^). The remaining minor adjustments to the training concept did not affect the analyses or the interpretation of our results and included factors such as the introduction of newly available games, which were not analyzed in this study.

One-way bivariate correlation analyses were conducted to investigate the relationship between game metrics and performance in clinical assessment within the corresponding neurocognitive subdomains. Depending on the distribution of the data, either Pearson (parametric statistical analyses) or Spearman (nonparametric statistical analyses) correlation analyses were conducted. *P* values and correlation coefficients (Pearson *r* or Spearman ρ), including 95% CIs, were calculated (using bootstrap for nonparametric analyses) [85]. To confirm the alternative hypothesis, we predetermined the following criteria: (1) statistically significant correlation (*P*≤.05; uncorrected; 1-sided) with (2) a correlation coefficient of ρ≥0.4, as proposed by Streiner et al [86] for studies in which perfect correlations are rare and many influencing factors are outside of the researchers’ control, which is the case in this analysis due to the complex nature of exergames and various influences such as task design, external factors, and personal characteristics. The correlation coefficients were interpreted as weak (ρ<0.3), moderate (ρ=0.3-0.5), or strong (ρ>0.5) [85,87].

We decided against applying a Bonferroni correction routinely for multiple comparisons considering our hypothesis and methodology. This is recommended because our study did not imperatively require the avoidance of type-I errors and we had preplanned hypotheses [88]. In addition, correlations were assessed only within their predefined neurocognitive subdomains, making our data dependent. Sullivan and Feinn [89] noted that applying the Bonferroni correction to dependent data may hinder the detection of relevant associations and increase the risk of a type-II error.

In this study, we did not conduct an a priori sample size calculation as we analyzed existing datasets from studies conducted as part of the Brain-IT project. Therefore, the sample size for these analyses was determined based on the number of datasets available from the 2 RCTs and was fixed and could not be altered. To ensure appropriate interpretation of our results, we conducted a post hoc power analysis using G*Power (version 3.1) [90,91]. As the Spearman rank correlation coefficient is computationally identical to the Pearson product-moment coefficient, power analyses were conducted using the same methodology as for estimating the power of a Pearson correlation for nonparametric analyses.

#### Secondary Objective: Longitudinal Analysis

For the secondary analysis, we only used game metrics that met the predefined criteria of the primary objective to ensure the validity of this analysis. For this analysis, data were collected longitudinally over the 12-week intervention period, excluding participants who dropped out during the intervention. The definition of each of our 10 game levels followed the principles described in the extended taxonomy supporting motor-cognitive learning by Huber et al [72]. As this taxonomy specifically integrates the model of skill acquisition [73] that has also been described to apply to cognitive skill learning and relearning [75], we based our longitudinal analysis on the model of skill acquisition [73], which integrates the 3-stage model of human skill acquisition by Fitts [74].

Accordingly, the performance data (game metrics) were presented as a function of time. As participants did not play each game an equal number of times, we expressed the variable “time” as a percentage of game completion time (ie, the first time that a participant played the game was 1%, and the last time they played the game was 100%). Full completion (100%) represented either the introduction of a new, slightly more challenging game or the end of the intervention. In addition, a second graph was generated displaying the performance changes within specific game levels. This was done to provide further insights into the data and is particularly relevant for accuracy metrics (eg, precision score), for which we expected different characteristics of the performance curves. More specifically, we generally expected either the typical sigmoid curve or curvilinear curves of rapid initial improvements followed by a plateau in performance. However, for accuracy metrics, we expected that this curve would only be visible within a specific game level, with a slight drop in accuracy each time the game level was increased.

Performance change curves were visually represented for each individual participant along with an overall curve that reflected the average performance across all participants [92]. However, we refrained from making any a priori assumptions on the type of relationships (eg, linear vs curvilinear relationship) as, on the one hand, our algorithmic decisions on individualized progression of exergame demands aimed to “break through” the expected (repeated) plateau in performance, which would be expected to create repeated curvilinear curves with a slight drop in performance after each plateau once the game difficulty was increased; however, on the other hand, there are no previous analyses available for the exergame systems and metrics used to base this theory-derived assumption on, whereas similar previous research has observed a more linear progression change over time in patients with mNCD in contrast to the typical curvilinear curves observed in healthy older adults [57]. Therefore, we chose to use the locally estimated scatterplot smoothing regression model to fit smooth curves, which provides a flexible approach to visualize data. This model is recommended for exploratory analysis because there is no need for a priori specification of relationships [93]. Finally, we measured and reported the average interquartile ranges number of times that participants played the game.

## Results

### Overview

**Figure 1:**
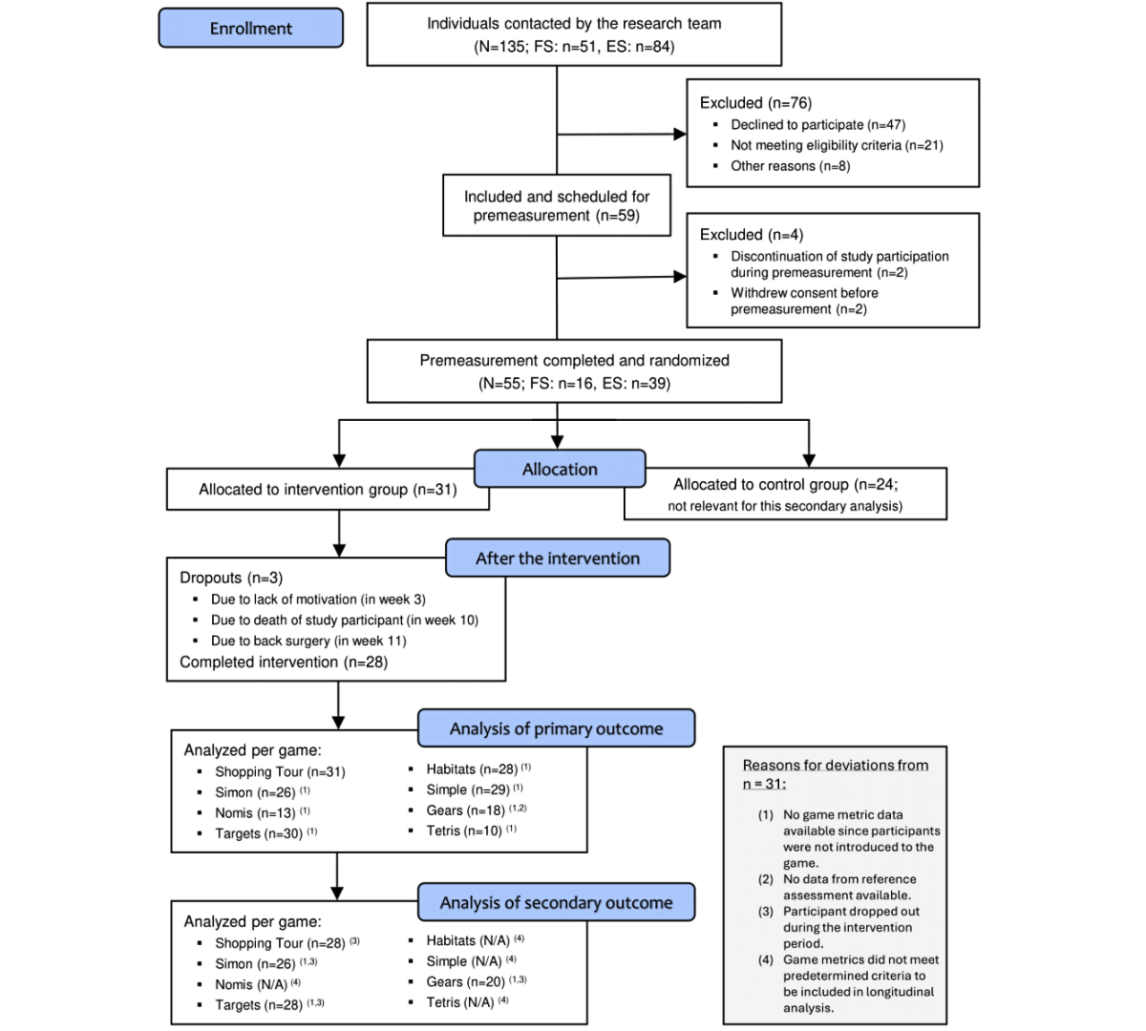
Summary of the participant flow throughout the study. FS: feasibility study; ES: effectiveness study; N/A: not applicable.

A summary of the participant flow is provided in Figure 1. Data were available for a total of 31 participants who were allocated to the intervention group in the 2 RCTs analyzed. The time between the initial premeasurement with the group allocation and the start of the intervention ranged from 1 to 2 weeks.

### Demographic Data

The demographic and clinical characteristics of all participants are listed in Table 2.

**Table 2:** Demographic and clinical characteristics of the participants in the intervention groups (N=31)^a^.

Participant characteristic			Values
Age (y), mean (SD)			76.4 (7.5)
Sex (female), n (%)			9 (29)
Education (y), mean (SD)			14.9 (4.0)
BMI (kg/m^2^), mean (SD)			24 (2.4)
Global cognitive performance (QMCI^b^ total score), mean (SD)			57.4 (14.0)
**Etiology of mNCD^c^, n (%)**			
	mNCD due to Alzheimer disease	19 (61)	
	Mild frontotemporal NCD^d^	4 (13)	
	Mild vascular NCD	5 (16)	
	Unclear or not yet determined	3 (10)	

^a^Demographic information was assessed based on self-report of the study participants. Global cognitive performance was assessed using the validated German version [66] of the Quick Mild Cognitive Impairment screen total score [77,78]. The characterization of the etiology of mild neurocognitive disorder was derived from the diagnostic information provided by the collaborating recruitment partners.

^b^QMCI: Quick Mild Cognitive Impairment screen.

^c^mNCD: mild neurocognitive disorder.

^d^NCD: neurocognitive disorder.

### Primary Objective: Cross-Sectional Analysis

Table 3 presents the results of the correlation analyses between game metrics and neurocognitive reference assessments. In the neurocognitive domain of learning and memory, 3 metrics of the game Shopping Tour (ie, mean reaction time, precision score, and collected items) and 1 metric of the game Simon (ie, point rate) met the alternative hypothesis criteria by exhibiting statistically significant moderate to strong correlations with the corresponding reference assessments (see Table 3 for all *P* values). In the neurocognitive domains of executive function and visuospatial skills, the metric point rate of the games Targets and Gears met the alternative hypothesis criteria by exhibiting statistically significant moderate correlations with the corresponding reference assessments. None of the metrics of the games targeting the neurocognitive domain of complex attention or of the remaining 2 games, Nomis and Tetris, met the alternative hypothesis criteria. Descriptive statistics for all outcome variables are provided in Tables S2 and S3 in Multimedia Appendix 1.

**Table 3:** Correlation between game metrics and reference assessments within their neurocognitive subdomains (N=31)^a^.

Neurocognitive domain, game and reference assessment, and outcome				Sample size, n (%)		Statistics										
						Type of analysis		*P* value		Spearman ρ (95% CI)		Pearson *r* (95% CI)		H_A_^b^ met?		Statistical power (post hoc)
**Learning and memory**																
	**Shopping Tour (WMS-IV-LM 1^c^ score)**															
		Mean reaction time (ms)	31 (100)		Nonparametric		<.001^d^		–*0.747* ^e^ (–0.876 to 0.548)		—^f^		Yes		0.979	
		Number of mistakes	31 (100)		Parametric		.15		—		–0.264 (–0.565 to 0.100)		No		0.669	
		Number of collected items	31 (100)		Nonparametric		<.001^d^		*0.691* (0.478 to 0.830)		—		Yes		0.923	
		Precision score (%)	31 (100)		Parametric		<.001^d^		—		*0.607* (0.374 to 1)		Yes		0.739	
	**Simon (PEBL^g^ DSF^h^score)**															
		Mean reaction time (ms)	26 (84)		Nonparametric		.04^d^		–0.344 (–0.680 to 0.145)		—		No		0.516	
		Point rate	26 (84)		Parametric		.008^d^		—		*0.471* (0.166 to 1)		Yes		0.528	
**Executive function**																
	**Nomis (PEBL DSB^i^ score)**															
		Mean reaction time (ms)	13 (42)		Nonparametric		.40		–0.079 (–0.575 to 0.572)		—		No		0.505	
		Point rate	13 (42)		Nonparametric		.60		–0.081 (–0.590 to 0.579)		—		No		0.705	
	**Targets (HOTAP combined score)**															
		Number of hits	30 (97)		Nonparametric		.03^d^		0.353 (–0.062 to 0.626)		—		No		0.514	
		Number of misses	30 (97)		Nonparametric		.08		–0.264 (–0.597 to 0.098)		—		No		0.512	
		Point rate	30 (97)		Nonparametric		.006^d^		*0.455* (0.144 to 0.715)		—		Yes		0.523	
**Complex attention**																
	**Habitats (TAP^j^ Go-No go reaction time)**															
		Mean reaction time (ms)	28 (90)		Parametric		.25		—		–0.131 (–1 to 0.195)		No		0.506	
		Point rate	28 (90)		Nonparametric		.36		0.072 (–0.371 to 0.448)		—		No		0.503	
	**Simple (PEBL TMT**-**A^k^ completion time)**															
		Mean reaction time (ms)	29 (94)		Nonparametric		.58		0.039 (–0.361 to 0.474)		—		No		0.719	
		Point rate	29 (94)		Nonparametric		.92		–0.264 (–0.541 to 0.079)		—		No		0.997	
**Visuospatial skills**																
	**Gears (PEBL MRT^l^ performance score)**															
		Mean reaction time (ms)	18 (58)		Nonparametric		.63		0.085 (–0.402 to 0.663)		—		No		0.752	
		Point rate	18 (58)		Nonparametric		.02^d^		*0.474* (0.018 to 0.787)		—		Yes		0.520	
	**Tetris (PEBL MRT performance score)**															
		Point score	10 (32)		Nonparametric		.06		–0.043 (–0.783 to 0.089)		—		No		0.069	

^a^The categorization of exergames into the neurocognitive domains and subdomains was derived from the published Brain-IT training concept, and for each game, we selected the most appropriate reference assessment from the available published datasets (Table 1). The variables listed in the first column describe all game metrics that were computed by the exergame system and, therefore, available for analyses (ie, no selection or exclusion of specific metrics).

^b^H_A_: alternative hypothesis.

^c^WMS-IV-LM 1: Wechsler Memory Scale–Fourth Edition–Logical Memory subtest part 1.

^d^Statistically significant at *P*<.05.

^e^Italics indicate a correlation coefficient of >0.4.

^f^Not applicable.

^g^PEBL: Psychology Experiment Building Language.

^h^DSF: digit span forward.

^i^DSB: digit span backward.

^j^TAP: Test of Attentional Performance.

^k^TMT-A: Trail Making Test part A.

^l^MRT: Mental Rotation Task.

### Secondary Objectives: Longitudinal Analysis

**Figure 2:**
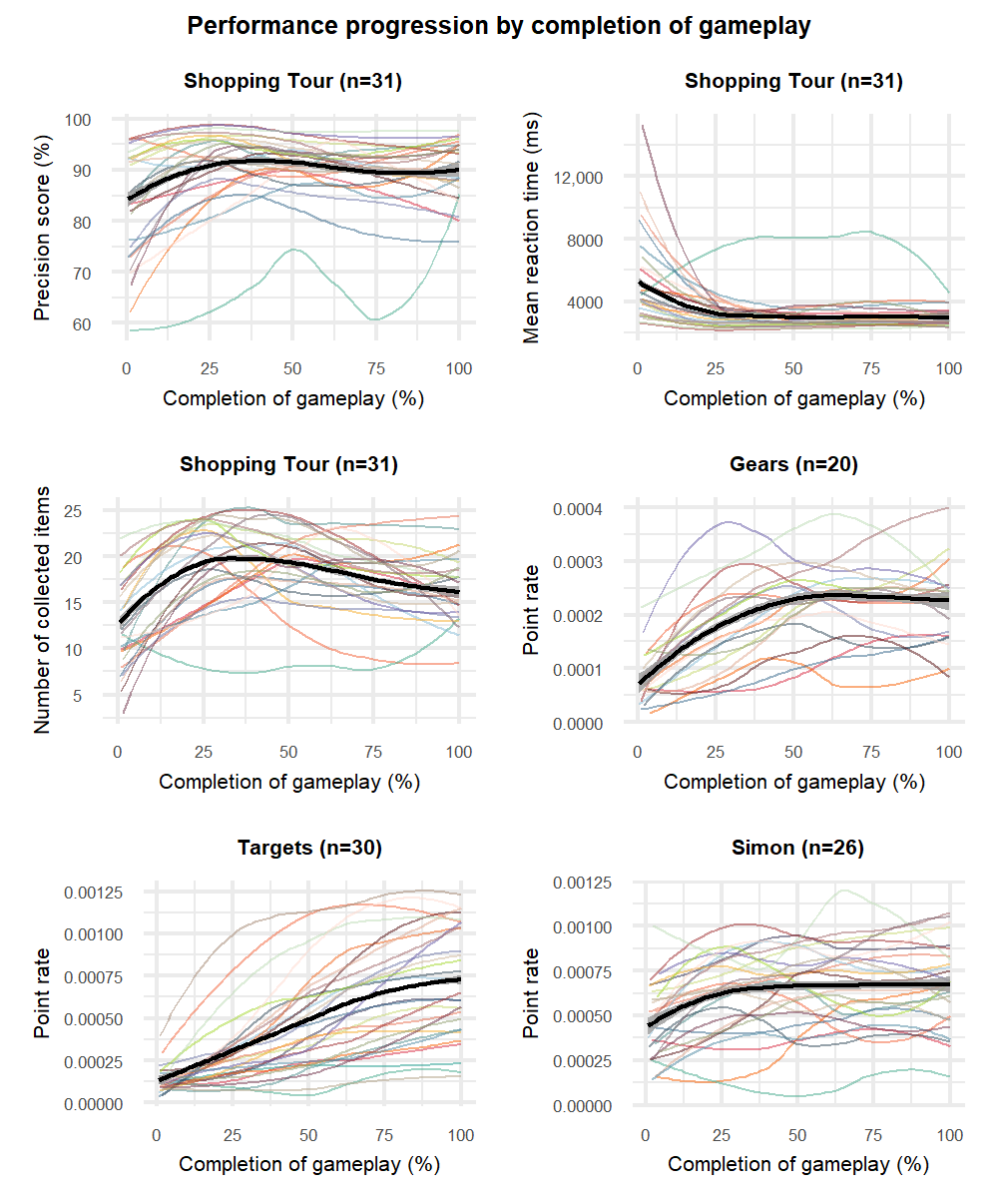
Performance progression over gameplay time.

**Figure 3:**
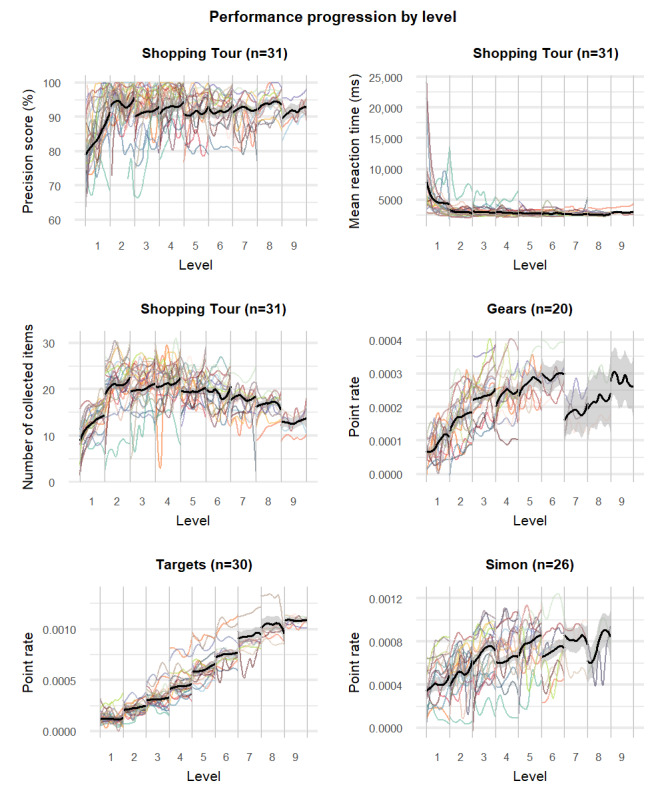
Performance progression over difficulty levels.

This analysis included the 6 game metrics that met the criteria for the primary objective. Table 4 presents the results for the number of times that the participants played each game. Figure 2 shows the performance progression curves over gameplay time in percentages. For all game metrics, there was an initial increase in average performance progression, or a decrease in the case of the metric “mean reaction time.” This was followed by a plateau (see Simon, Targets, and the mean reaction time metric of Shopping Tour) or even a slight decline (see Gears and the precision score and collected items metrics of Shopping Tour) in performance. Interindividual differences in performance progression were observed, particularly for the metric “point rate.” While some individuals exhibited a flatter curve, others had a more pronounced initial increase or a sigmoidal curve pattern. Figure 3 shows the performance progression within difficulty levels. In addition to the findings shown in Figure 2, we observed that, in the game Shopping Tour, the metrics “precision score” and “collected items” increased within most levels, whereas the metric of mean reaction time initially declined and then leveled off. For the metric of point rate in the games Gears and Simon, we observed considerable drops in performance between certain levels of difficulty.

**Table 4:** Sessions played per participant that were analyzed for the secondary objective^a^.

Game	Sessions played, median (IQR)
Shopping Tour	132 (99)
Simon	72 (23)
Targets	95 (44)
Gears	64 (25)

^a^The descriptive statistics presented refer to the median and the IQR of the number of sessions that the study participants (individuals with mild neurocognitive disorder) played in each of the exergames analyzed in this study over the 12-week intervention period.

## Discussion

### Principal Findings

#### Overview

This study aimed to identify valid game metrics as indicators for in-game domain-specific cognitive performance during exergaming in individuals with mNCD by analyzing the correlation between game metrics and neurocognitive assessments. Key findings were that 33% (6/18) of the game metrics within the neurocognitive domains of learning and memory, executive function, and visuospatial skills were identified as valid indicators for in-game domain-specific cognitive performance during exergaming. This study’s secondary objective was to visually explore game metric performance changes over time and within difficulty levels. The results revealed high interindividual variability and overall trends of the expected typical curvilinear curves of rapid initial improvements followed by a plateau in performance.

#### Primary Outcome: Cross-Sectional Analysis

In total, 4 games (Shopping Tour, Simon, Targets, and Gears) had at least one metric that met the predefined criteria and, therefore, was identified as a valid indicator for in-game domain-specific cognitive performance during exergaming. We observed that 83% (5/6) of the game metrics that met our alternative hypothesis reflected the precision of response (ie, point rate or precision score), whereas measures of the speed of response (ie, mean reaction time) met the criteria in only 17% (1/6) of cases even in the neurocognitive domain of “complex attention,” which specifically trains processing speed. Furthermore, the FS of the Brain-IT training concept has already shown that reaction times had a high inter- and intraindividual variability, making it difficult to initiate training adaptations based on these game metrics [58]. These observations suggest that game metrics reflecting the precision of response may be a better indicator of cognitive performance during exergaming than metrics reflecting the speed of response. The game Shopping Tour exhibited strong correlations in 75% (3/4) of the metrics, whereas other games exhibited weak correlations in all collected metrics. This suggests that the results may depend on two main factors: (1) the validity of game metrics as an indicator of performance and (2) how well the game content aligns with its assigned neurocognitive domain or subdomain. The second factor, the content validity of the game, was confirmed by neuropsychologists during the design phase of the Brain-IT training concept [61]. However, it is important to note that exergames typically target different neurocognitive functions as well as motor skills. Although each game focuses on a specific neurocognitive domain or subdomain, other domains are often indirectly trained, which may confound the domain-specific analyses. Therefore, the observation that certain games displayed no metric that met the alternative hypothesis criteria is likely related to the validity of the game metrics as indicators of performance or to the content of the games not being specific enough to target the corresponding neurocognitive domains or subdomains.

While we have established that game metrics and game content may contribute to measuring domain-specific exergame performance, the complex nature of exergames indicates that additional factors more closely related to the training itself should also be considered. These factors are typically minimized in clinical assessments, yet they are a significant aspect of exergaming and may have influenced the outcomes of this study. An important consideration is the influence of the exergame design, including both hardware and software, on player performance. One such task design aspect is the scoring system. If certain tasks within the game are not appropriately scored relative to their difficulty, this could result in an inaccurate reflection of performance measures. Another consideration is the involvement of different difficulty levels. In our analysis, we correlated game metrics from only the first level of difficulty, which was considered an introductory level and may not have been challenging enough to show the full capabilities of the participants. In line with this consideration, Litz et al [94] conducted a similar study correlating exergame metrics with clinical assessments but across various difficulty levels and found some variable outcomes for the same game in different levels, with a tendency toward more consistent relationships between in-game metrics and standardized cognitive assessments at higher difficulty levels or when averaging scores over all game levels. Furthermore, exergames often include feedback mechanisms that reward correct decision-making (ie, precision of response) more than decision speed, which may explain the low correlation with reaction time metrics in our results. External factors such as physical environment or external support, as well as personal factors such as emotional status or self-efficacy [95], should also be considered as potential influencers of performance. In relation to this, Sajjadi et al [49] conducted a systematic review on adapting serious games to users with the aim of increasing engagement and effectiveness. This review proposed the use of game metrics for in-game adaptions but also highlighted the importance of considering additional factors such as physiological states and the personal traits of the user [49].

Our findings align with those of previous studies that have analyzed correlated metrics from exergames with clinical assessments of cognitive performance [54,56,57,94]. Specifically, Guimarães et al [57] correlated game metrics measured on the first gameplay against standard cognitive and mobility assessments, including the Montreal Cognitive Assessment (MoCA), in 13 older adults with mobility limitations. They observed that 2 of the 16 analyzed game metrics (from 4 different games) showed a statistically significant correlation with the MoCA total score, both related to scores on precision or accuracy of responses. They also correlated these game metrics with all the MoCA subdomain scores and found some (ie, 8 of the 112 analyses) statistically significant correlations with the visuospatial, executive functioning, and abstraction components of the MoCA. Similar results were observed for the physical performance outcomes, with several (17 of the 48 analyses) step characteristic outcomes during exergaming showing statistically significant correlations with physical test scores [57]. Petsani et al [54] computed Pearson correlations between the in-game metrics of 13 individuals with Parkinson disease with the Symbol Digit Modalities Test using a feature vector containing the mean value of all sessions for each in-game metric for each participant. They observed that 1 of the 11 analyzed game metrics (from 4 different games) showed a strong correlation with the Symbol Digit Modalities Test score [54]. Konstantinidis et al [56] computed Pearson correlations between an aggregated score derived from various in-game metrics of 113 participants (ie, 38 cognitively healthy, 64 with mild cognitive impairment, and 14 with mild dementia) with the Mini-Mental State Examination (MMSE), MoCA, and Trail Making Test parts A and B. They found consistent, statistically significant, moderate to strong correlations for all 4 analyses [56]. Finally, Litz et al [94] computed Spearman rank correlations between in-game metrics and 5 outcomes from 4 cognitive assessments in independently living older adults who were cognitively intact and multimorbid. They observed consistent moderate to strong correlations for most (88 out of 130) analyses, with similar observations for a range of physical assessments [94].

These observations indicate that only a selection of game metrics may be valid indicators for in-game cognitive performance. However, identifying specific game metrics that performed better across these different studies was challenging due to the high heterogeneity in study design, analyzed game metrics, and reference assessments used. Notably, while our study was based on a domain-specific approach, these previous studies correlated game metrics with a range of cognitive (and physical) assessments without initial categorization of game specificity. In addition, they frequently used broad assessments, such as the MoCA and MMSE, which are tests that cover several cognitive domains in the outcome score [96,97]. The correlation analyses between different game metrics and the MoCA or MMSE ranged from weak [57,94] to moderate [56,57] and strong [56,57], indicating diverse outcomes. When investigating specific neurocognitive domains, we also found diverse findings. In contrast to our findings, Litz et al [94] found moderate to strong correlations for the metrics scores and reaction time within the domain of complex attention. On the other hand, we found some moderate to strong correlations within the domain of learning and memory, whereas Guimarães et al [57] did not find such correlations in similar games.

Some studies have also used game metrics to predict cognitive or physical performance as measured using standardized clinical assessments. Petsani et al [54] used a decision tree classifier with the Gini index to predict, based on in-game metrics, whether participants belonged to groups with better or worse physical and cognitive states. This categorization was derived from clustering analysis based on 18 outcomes from a set of neuropsychological and physical assessments. They observed a high classification accuracy at 84.6% [54]. Konstantinidis et al [56] used a multilayer feedforward neural network to assess the predictive value of their aggregated game score and found an overall accuracy of 70.69% to distinguish between cognitively healthy, mild cognitive impairment, and mild dementia [56].

In summary, previous studies have shown mixed results but were able to identify certain game metrics that appear as valid indicators for physical, cognitive, or motor-cognitive performance in exergaming and may be reflective of standardized neuropsychological or physical assessments. However, while the use of such game metrics to individually tailor and adjust the games is widespread, these metrics have evidently rarely been scientifically investigated as we only identified a handful of similar previous studies [54,56,57,94]. Therefore, it appears important to carefully select and prevalidate game metrics in future studies, especially when they are used for purposes such as in-game adaptations or performance monitoring. Given the current advancements in computing fields such as artificial intelligence, it is likely that real-time personalized adaptation will play an even more important role in the future of exergames [98]. This development requires valid metrics as indicators of physical and cognitive in-game performance characteristics. The challenge for researchers and game developers is to identify or develop valid game metrics and integrate them strategically into exergame designs, preferably with a strong background in sports science or neuroscience to ensure construct validity, as previously proposed by our research group [58].

#### Secondary Outcome: Longitudinal Analysis

The results of the secondary objective indicated a common trend across all games, with the expected typical curvilinear curves of rapid initial improvements suggesting an early phase of skill acquisition followed by a plateau in performance. This finding is consistent with those of the existing literature [74,99,100] as well as with models of skill acquisition [73,74] that have also been described to apply to cognitive skill learning and relearning [75]. Specifically, the model of human performance by Fitts and Posner [74] identifies 3 stages: the cognitive phase, in which the user learns what to do and experiences a rapid performance gain; the associative phase, in which the user refines their actions and increases efficiency; and, finally, the autonomous phase, in which the skill becomes automatic and the user becomes proficient.

In our analysis, certain game metrics exhibited a more pronounced plateau (ie, Simon) or even a decline in performance (ie, Gears and the collected items metric of Shopping Tour), which would not be expected from the literature and suggests that players may not have experienced the associative phase. However, upon examining the data across specific difficulty levels, we observed that these plateaus did not necessarily indicate a halt in performance improvement but, rather, an increase in task difficulty that was not appropriately rewarded by the game metrics. In some games, the task difficulty significantly increased between certain levels (eg, Gears with more complex figures or Simon with longer sequences to remember; see the published training concept in Supplementary File 2 of the ES [59]). Between these levels of increasing task difficulty, a clear drop in performance was observed (eg, in Gears after levels 3 and 6 or in Simon after levels 1, 3, 5, and 7). This indicates that, for these games, the scoring system failed to account for varying levels of difficulty by awarding the same number of points for correctly completed tasks of varying difficulty. In contrast, the performance progression of Targets and the precision score metric of Shopping Tour demonstrated a consistent improvement with individual dips and leaps as is expected [101]. This indicates that the adaptation mechanisms were functioning well for these games and the game metrics can be used to monitor performance progression. Furthermore, for the game Shopping Tour, we observed inconsistent curves for different metrics. The mean reaction time decreased as the game level increased, reached an early plateau, and remained low. This was a somewhat unexpected finding as we expected reaction times to increase slightly and then show the typical reciprocal curve with repeated practice within the same game level, similar to what we expected from the precision metric. This observation suggests that average reaction speed may play a subordinate role in this game. In contrast, the precision score showed continuous improvement, with the expected temporary drops immediately after increasing the game levels (with the exception of the increase from level 1 to 2), which was similar to what was found in the study by Guimarães et al [57]. The exceptional observation that the precision score in the game Shopping Tour did not temporarily drop when the game level increased from level 1 to level 2 may be explained by (1) early habituation and learning effects in the use of and interaction with the exergame device and the specific game and (2) the fact that the increment between these levels may have been too small to induce a temporary drop in performance.

Overall, these observations may indicate that measures of precision of response are also better in reflecting performance changes over time compared to measures of speed of response. In general, both visual representations of performance progression (over gameplay time in Figure 1 and across difficulty levels in Figure 2) provided valuable insights. If the scoring system can account for varying levels of difficulty (with scores that increase with task difficulty), the first option (Figure 1) may be recommended because it enables the analysis of an individual’s overall performance progression regardless of the difficulty level they finally reached. However, this approach does not apply to accuracy metrics (eg, precision score). Accuracy metrics should exhibit a plateau curve toward maximum precision followed by a sharp initial drop after each level increase in the game followed by the typical reciprocal curves of improvement followed by a plateau in performance. This pattern is consistent with the results obtained in this study. However, it can only be observed in the visual representation that shows different levels of difficulty (Figure 2). In summary, our findings indicate the need for adaptable game metrics that can also account for varying tasks and levels of difficulty within the game.

Our data also demonstrate high interindividual variability in performance progression. These findings could be associated with the high variability in adherence (total sessions played per participant), which was mainly because some participants played the games much more often than instructed. In particular, the total number of completed sessions ranged from 33 to 159, with an average number of completed sessions of 54.4 (SD 13.0) in the FS and 71.5 (SD 26.2) in the ES [58,59]. However, it could also be associated with clinical or demographic characteristics of the participants. Research does confirm that individuals adapt their skills at different rates, which might be attributed to different learning styles [92,99,102]. Furthermore, previous studies have indicated that cognitively impaired groups have been observed to exhibit a flatter performance progression with a less prominent initial increase compared to healthy controls [57,103]. Other studies have suggested that age has an influence on individual performance progress in serious games [104,105]. Therefore, the observed interindividual variability in our analysis could be considered normal and an expected outcome as our group was relatively heterogeneous in clinical and demographic characteristics. Additional research is required to explore what individual characteristics may lead to different performance progressions; identify predictors for steeper performance curves in this field; and apply these findings to identify and adopt strategies that facilitate individual learning success and, thus, effectiveness in improving cognitive (and physical) performance.

### Strengths and Limitations

One of the main strengths of this study was that we conducted the correlation analyses between game metrics and clinical assessments only within specific preassigned neurocognitive domains or subdomains. This approach was enabled by the already available content validation of the games to their primary trained neurocognitive domain or subdomain within the Brain-IT training concept [61]. Therefore, the primary objective was hypothesis driven, with predefined criteria for interpretation of the results. In addition, we used data from clinically validated and well-established neuropsychological assessments across all neurocognitive domains.

There are also some limitations that need to be discussed. First and most importantly, the sample size was small, and we did not conduct an a priori sample size calculation as we analyzed existing datasets from studies conducted as part of the Brain-IT project. To ensure appropriate interpretation of our results, we conducted a post hoc power analysis using G*Power (version 3.1) [90,91], which revealed that most of our analyses were underpowered. This might have resulted in potential false-negative or false-positive results, affects the robustness and generalizability of our findings, and warrants confirmatory studies with a priori sample size calculations to ensure more reliable and robust conclusions. Considering our criteria to confirm the alternative hypotheses as assumptions for a power analysis, a sample size of 37 or 50 would be required to show (1) a statistically significant correlation (*P*≤.05; uncorrected; 1-sided) with (2) a correlation coefficient of ρ≥0.4 with sufficient power of β>.80 or β>.90, respectively, which may be used as a reference for planning future studies on this topic.

Second, this study was a secondary analysis, and the original studies as well as the intervention were not originally designed a priori to investigate the objectives of this study, which restricted our methodological choices in this secondary analysis. Although the game sessions were standardized in terms of game design and intervention delivery, there were interindividual differences in gameplay due to personalization and individual progression of the intervention.

Specifically, these restrictions limited the following methodological aspects for the primary research question. According to the Brain-IT training concept, the games were individually assigned considering the participants’ baseline neuropsychological performance, with all participants starting at level 1 of the game demands in the first training session. Thereafter, either the games progressed or more difficult games were introduced as soon as (1) a plateau in performance was reached on predetermined game metrics, (2) a predetermined target score was reached on a predetermined game metric for progression to the next level, (3) the participant requested an increase in task demands, or (4) the staff supervising the participants deemed the increase in task demands feasible [59]. Given these rules for individualized progression of exergame demands, the path through these games and game levels was different for each participant, with large differences in the time to progress to the next levels and the maximum level reached after the 12-week intervention (as shown in Figure 3). For example, one participant might reach level 5 after only 2 weeks of training, whereas another might not reach this level until after 12 weeks. As the first research objective was tied to baseline cognitive performance as measured using standardized neuropsychological assessments, only the first level provided the same standardized conditions for all study participants that allowed the cross-sectional analyses to address this research objective. This methodological limitation may have resulted in participants with higher baseline functioning being underchallenged. However, we did not observe any floor or ceiling effects in any of the game metrics except for number of misses in the game Targets and point score in the game Tetris (Figures S1-S8 in Multimedia Appendix 1), whereas there was a good amount of variability in all game metrics (Table S3 in Multimedia Appendix 1). Therefore, it can be assumed that this limitation did not have a significant impact on the observed results. Nevertheless, analysis of higher levels of difficulty would undoubtedly provide additional insights. As an example, the findings of Litz et al [94] observed a tendency toward more consistent relationships between in-game metrics and standardized cognitive assessments at higher difficulty levels or when averaging scores over all game levels [94]. However, given the individual paths through the game levels and the corresponding time differences to reach a given level in this study, it would be impossible to disentangle learning effects (for participants who had already played the game several times) and performance improvements over time during the intervention from “true” cognitive performance as measured at baseline. Furthermore, not all participants played the same games, resulting in smaller sample sizes in some games. Games that were considered more difficult, such as Nomis and Tetris, had small sample sizes as they were only played by participants with a higher baseline cognitive performance in the neurocognitive domains of executive functioning and visuospatial skills, which informed the allocation of the games in the first session. This could have biased the analysis. Therefore, future studies should be designed to specifically address the research question of this study rather than relying on existing datasets that might restrict the methodological choices in the analyses of the data. This entails a cross-sectional design with a priori sample size calculations rather than the use of data from longitudinal studies and applying different game levels to investigate whether the game levels induce the appropriate changes in perceived task demands (eg, as has been investigated in the work by Manser and de Bruin [71]) and whether and how performance at higher difficulty levels might differentially relate to cognitive performance assessed using standardized neuropsychological assessments.

The personalization and individualized progression of the training also presented a challenge for the analysis of our secondary research question (ie, performance progression) as players varied in the number of times they played the games and difficulty levels were individually adapted. To account for these interindividual differences, we found a method to present performance progression across gameplay by normalizing time to game completion as a percentage. Using this method, we were able to account for variability in adherence and difficulty levels at a group level. However, this method may result in loss of some information, and individual curves were compressed or stretched. These methodological constraints also prevented us from conducting more in-depth analyses of the observed high interindividual variability in performance progression (see Figures 2 and 3 with color coding of individual participant progression curves), such as exploratory analyses to identify potential factors contributing to the variability (eg, age, years of education, and baseline cognitive status) as these factors (particularly baseline cognitive status) influenced individualized tailoring (personalized allocation as well as individualized progression of games and game levels). Therefore, future studies should be specifically designed to account for these aspects and allow for more in-depth analysis to disentangle the factors that influence the high variability in performance progression over time and derive evidence-based recommendations on the implications of accounting for this variability in the methodological aspects of individually tailoring exergame-based training.

Third, although each exergame focused on 1 specific neurocognitive domain or subdomain, they often indirectly trained other domains, which may have confounded the domain-specific analysis. Fourth, the absence of data from specific motor assessments precluded their inclusion in our analyses. Finally, this study only included older adults with mNCD, and the results cannot be generalized to other populations.

### Implications for Research

The personalization of training is becoming increasingly popular in the current development of technology-supported interventions, including exergames. Within this field, research should aim to identify valid parameters that can be used for in-game adaptation. The potential of game metrics should be further investigated through studies with structured interventions while also considering the impact of game task design as well as personal and external factors. Given the proposal by Netz [106] that (adherence to) optimal exercise intensity and motor-cognitive task complexity are driving mechanisms for global and task-specific neuroplasticity, respectively, and as discussed in previous research, personalized training adaptations should go beyond game metrics and consider additional aspects of players related to physiological states (e.g., attention, stress) or personal characteristics (e.g., learning style, intelligence) to provide an optimal exercise intensity and motor-cognitive task complexity [49]. Different biofeedback systems, such as biocybernetic adaptation loops, which continuously adjust game difficulty or game content in response to real-time physiological data from the user [107-109], are currently being investigated in this area and should be further explored. Furthermore, previous reviews have identified several features of fully immersive virtual reality (VR) that can support skill learning [110], whereas fully immersive VR or augmented reality has a fundamental advantage in delivering ecologically valid game scenarios via recreating a certain level of naturalistic sensory-motor interaction between the user and graphical user interface [111]. Such features include but are not limited to multisensory interactivity with haptic feedback in a high-fidelity VR with 3D representations and the presence of avatars [110]. Given that specially designed and customized VR interventions have been more effective in neurorehabilitation compared to the use of commercially available VR systems [112], these features should be more thoroughly investigated regarding their capacity to improve tailoring of the exercises to provide optimal exercise intensity and motor-cognitive task complexity.

The variability in performance progression across participants presents further aspects that need to be investigated. Research should try to identify factors that can help understand and identify how individuals differ in their cognitive and motor-cognitive skill acquisition and performance progression. Different factors that may contribute to a steeper progression curve should be investigated. This should include subgroup analyses, which we suggest should include assessing (1) the baseline physical and cognitive state of the individuals [57,103,105], (2) age [104,105], (3) adherence, and (4) self-efficacy [95]. Moreover, game metrics obtained in the “game module” only provide information on physical and cognitive performance in a specific task (game). However, solely analyzing game metrics does not allow for any conclusions on the efficacy or effectiveness of the training as the habituation and learning effects cannot be disentangled from “true” physical and motor-cognitive performance improvements. Such “true” performance adaptations would either be assessed using standardized physical, motor, or neuropsychological assessments or obtained in a separate evaluation module that integrates scientifically validated gamified assessments to regularly verify whether the exercise or training stimulus is sufficient to induce the desired skill-related changes or near- and far-transfer effects. Therefore, future longitudinal studies should also seek to investigate to which extent performance improvements measured using game metrics within the “game” module translate to physical, motor, or cognitive performance enhancements measured using validated assessments and elucidate factors for promoting such transference effects.

It has been proposed that (adherence to) optimal intensity and complexity of exercises are the driving mechanisms for global neuroplasticity (ie, exercise intensity) and task-specific neuroplasticity (ie, cognitive and motor-cognitive task complexity) [106]. The ability of technology-enhanced training to individually progress exercises according to performance metrics in real time offers a potential advantage over conventional exercises and is considered a key advantage of serious video games (such as exergames or cognitive training games) [113]. There is initial empirical evidence supporting this assumption by showing that training with adaptive difficulty algorithms may be superior to generic games to improve both cognitive and noncognitive outcomes in individuals with mNCD in the context of computerized cognitive training [114]. Most studies on exergame-based training [5,115] and technology-supported physical activity interventions in general [46] seem to also have recognized this potential benefit and individually tailored or progressed the exercises by adjusting the difficulty or complexity of the task or games according to the users’ ability or performance [115]. However, only 42% [5] or 14% [46] of studies have reported or provided sufficient details on exercise intensity, whereas details on cognitive demands were neither assessed nor reported in any of the analyzed studies in the review by Manser et al [5]. Moreover, while a recent review observed that tailored technology-enhanced physical activity interventions (including exergame-based training) resulted in significantly higher adherence than control interventions [46], a recent systematic review observed that the effects on cognitive performance were moderated not by individualization of training (ie, tailored vs generic [one size fits all]) for exergame-based training in middle-aged to older adults but by other variables (eg, “body position” variable, favoring stepping movements in a standing position, or “exercise intensity” variable, favoring moderate exercise intensity) [5]. Therefore, it remains to be investigated in more detail whether individualization of training translates to moderating the efficacy or effectiveness of technology-enhanced training and how such individualization may be optimally designed to maximize efficacy or effectiveness in exergame-based training [5], as well as the broader context of technology-enhanced physical and motor-cognitive training. Ultimately, these investigations should seek to improve the personalization of technology-enhanced interventions by identifying and adopting strategies that facilitate individual learning success and, thus, promote effectiveness in improving cognitive (and physical) performance.

### Conclusions

This secondary analysis of 2 RCTs provided valuable insights into exergame metrics. This study demonstrated that a selection of game metrics can serve as valid indicators of in-game domain-specific cognitive performance during exergaming. Moreover, metrics that reflect the precision of responses overall performed better than metrics reflecting the speed of responses, suggesting that metrics for precision may be better indicators of cognitive performance and performance progression during exergaming. Therefore, it is recommended to carefully select and prevalidate game metrics for purposes beyond game performance measurement and monitoring, such as informing personalized adjustments of the difficulty and complexity of exergames. Incorporating valid game metrics could increase their value in exergame designs, which, in turn, could increase exergame engagement and effectiveness. Due to the complex nature of exergames, in-game performance may depend on additional factors such as game design, external conditions, and personal characteristics of the player. Furthermore, the analysis of performance progression revealed high interindividual differences, underlining the importance of personalized training and suggesting the need for further research to explore characteristics explaining these differences and identify and adopt strategies that facilitate individual learning success and, thus, promote effectiveness in improving health- or disease-related outcomes.

## Appendix

Multimedia Appendix 1 CONSORT 2017 checklist for nonpharmacologic treatments and supplementary tables and figures, including descriptive statistics for clinical assessments and game metric data, along with scatterplots of correlation analyses between exergame metrics with neuropsychological assessments.

Multimedia Appendix 2 CONSORT-eHEALTH checklist (V 1.6.1).

## Abbreviations

CONSORT: Consolidated Standards of Reporting Trials
ES: effectiveness study
FS: feasibility study
MMSE: Mini-Mental State Examination
mNCD: mild neurocognitive disorder
MoCA: Montreal Cognitive Assessment
PEBL: Psychology Experiment Building Language
RCT: randomized controlled trial
VR: virtual reality

## Data Availability

Publicly available datasets for the feasibility study [116] and the effectiveness study [117] were analyzed in this study. These datasets include the demographic and clinical information analyzed, as well as the raw data for the cognitive assessments. Descriptive statistics on the data on the game metrics are included in this paper and its supplementary files. Further inquiries can be directed to the corresponding author.
