# Supplementary material for: Domain-Specific Evaluation of Exergame Metrics Among Older Adults With Mild Neurocognitive Disorder: Secondary Analysis of 2 Randomized Controlled Trials
Source: JMIR Serious Games. 2025 May 21;13:e65878. doi: 10.2196/65878 (PMC12138312; doi:10.2196/65878)
Supplement: Multimedia Appendix 1 [file games_v13i1e65878_app1.pdf]

CONSORT checklist

Table S1: 2017 CONSORT (Consolidated Standards of Reporting Trials) Checklist of Information to Include When Reporting Randomized Trials Assessing nonpharmacologic treatments.

Abbreviations: FS = Feasibility Study (Pilot RCT), ES = Effectiveness Study (RCT)

Section/Topic:	Item No:	Checklist item:	Reported in section(s):
**TITLE AND ABSTRACT:**			
	1a	Identification as a randomized trial in the title.	reported as a secondary analysis of two RCTs in 'Title'
	1b	Structured summary of trial design, methods, results, and conclusions (for specific guidance see CONSORT for abstracts).	'Abstract'
**INTRODUCTION:**			
Background and objectives:	2a	Scientific background and explanation of rationale.	'Introduction - Background'
	2b	Specific objectives or hypotheses.	'Introduction - Objectives'
**METHODS:**			
Trial design:	3a	Description of trial design (such as parallel, factorial) including allocation ratio. When applicable, how care providers were allocated to each trial group.	'Methods', 'Methods - Study Design and Participants', more details in FS [1] and ES [2]
	3b	Important changes to methods after trial commencement (such as eligibility criteria), with reasons.	Details in FS [1] and ES [2]
Participants:	4a	Eligibility criteria for participants. When applicable, eligibility criteria for centers and for care providers.	'Methods - Study Design and Participants', more details in FS [1] and ES [2]
	4b	Settings and locations where the data were collected.	'Methods - Study Design and Participants', more details in FS [1] and ES [2]
Interventions:	5	The interventions for each group with sufficient details to allow replication, including how and when they were actually administered. Precise details of both the experimental treatment and comparator.	'Methods - Training Intervention', more details in training concept (Supplementary File 3 from the methodology paper [3]), FS [1] and ES [2]
	5a	Description of the different components of the interventions and, when applicable, description of the procedure for tailoring the interventions to individual participants.	'Methods - Training Intervention', more details in training concept (Supplementary File 3 from the methodology paper [3]), FS [1] and ES [2]
	5b	Details of whether and how the interventions were standardized.	'Methods - Training Intervention', more details in training concept (Supplementary File 3 from the methodology paper [3]), FS [1] and ES [2]
	5c	Details of whether and how adherence of care providers to the protocol was assessed or enhanced.	N/A
	5d	Details of whether and how adherence of participants to interventions was assessed or enhanced.	'Methods - Outcomes', more details in FS [1] and ES [2]
Outcomes:	6a	Completely defined pre-specified primary and secondary outcome measures, including how and when they were assessed.	'Methods - Outcomes' and 'Methods - Data Analysis and Statistical Methods'

	6b	Any changes to trial outcomes after the trial commenced, with reasons.	N/A
Sample size:	7a	How sample size was determined. When applicable, details of whether and how the clustering by care providers or centers was addressed.	N/A (as documented in 'Methods - Data Analysis and Statistical Methods', sample size was defined data availability)
	7b	When applicable, explanation of any interim analyses and stopping guidelines.	not relevant for the secondary analysis, details in FS [1] and ES [2]
Randomization:			
Sequence generation:	8a	Method used to generate the random allocation sequence.	not relevant for the secondary analysis, details in FS [1] and ES [2]
	8b	Type of randomization; details of any restriction (such as blocking and block size).	'Methods - Study Design and Participants'
Allocation concealment mechanism:	9	Mechanism used to implement the random allocation sequence (such as sequentially numbered containers), describing any steps taken to conceal the sequence until interventions were assigned.	not relevant for the secondary analysis, details in FS [1] and ES [2]
Implementation:	10	Who generated the random allocation sequence, who enrolled participants, and who assigned participants to Interventions.	not relevant for the secondary analysis, details in FS [1] and ES [2]
Blinding:	11a	If done, who was blinded after assignment to interventions (e.g., participants, care providers, those administering co-interventions, those assessing outcomes) and how.	not relevant for the secondary analysis, details in FS [1] and ES [2]
	11b	If relevant, description of the similarity of interventions.	N/A
	11c	If blinding was not possible, description of any attempts to limit bias	N/A
Statistical methods:	12a	Statistical methods used to compare groups for primary and secondary outcomes. When applicable, details of whether and how the clustering by care providers or centers was addressed.	'Methods - Data Analysis and Statistical Methods'
	12b	Methods for additional analyses, such as subgroup analyses and adjusted analyses	'Methods - Data Analysis and Statistical Methods'
**RESULTS:**			
Participant flow (a diagram is strongly recommended):	13a	For each group, the numbers of participants who were randomly assigned, received intended treatment, and were analyzed for the primary outcome. The number of care providers or centers performing the intervention in each group and the number of patients treated by each care provider or in each center.	'Results - Participants'
	13b	For each group, losses and exclusions after randomization, together with reasons.	'Results - Participants', more details in FS [1] and ES [2]
	13c	For each group, the delay between randomization and the initiation of the intervention.	'Results - Participants'
	13d	Details of the experimental treatment and comparator as they were implemented.	'Results - Participants', more details in FS [1] and ES [2]
Recruitment:	14a	Dates defining the periods of recruitment and follow-up.	not relevant for the secondary analysis, details in FS [1] and ES [2]
	14b	Why the trial ended or was stopped.	not relevant for the secondary analysis, details in FS [1] and ES [2]
Baseline data:	15	A table showing baseline demographic and clinical characteristics for each group. When applicable, a description of care providers (case volume, qualification, expertise, etc.) and centers (volume) in each group.	'Results - Demographic Data'

Numbers analyzed:	16	For each group, number of participants (denominator) included in each analysis and whether the analysis was by original assigned groups.	‘Results - Participants’
Outcomes and estimation:	17a	For each primary and secondary outcome, results for each group, and the estimated effect size and its precision (such as 95% confidence interval).	Primary Outcome: ‘Results - Primary Objective - Cross Sectional Analysis’ Secondary Outcome: ‘Results - Secondary Objective - Longitudinal Analysis’
	17b	For binary outcomes, presentation of both absolute and relative effect sizes is recommended	N/A
Ancillary analyses:	18	Results of any other analyses performed, including subgroup analyses and adjusted analyses, distinguishing pre-specified from exploratory	N/A
Harms:	19	All important harms or unintended effects in each group (for specific guidance see CONSORT for harms)	details in FS [1] and ES [2]
**DISCUSSION:**			
Limitations:	20	Trial limitations, addressing sources of potential bias, imprecision, and, if relevant, multiplicity of analyses. In addition, take into account the choice of the comparator, lack of or partial blinding, and unequal expertise of care providers or centers in each group.	‘Discussion - Primary Objective - Cross Sectional Analysis’; ‘Discussion - Strengths and Limitations’
Generalizability:	21	Generalizability (external validity) of the trial findings according to the intervention, comparators, patients, and care providers and centers involved in the trial.	‘Discussion - Strengths and Limitations’
Interpretation:	22	Interpretation consistent with results, balancing benefits - and harms, and considering other relevant evidence.	‘Discussion - Primary Objective’, ‘Discussion - Secondary Objective’
**OTHER INFORMATION:**			
Registration:	23	Registration number and name of trial registry.	‘Methods - Study Design and Participants’
Protocol:	24	Where the full trial protocol can be accessed, if available.	‘Methods’
Funding:	25	Sources of funding and other support (such as supply of drugs), role of funders.	‘Declarations - Funding’

Supplementary Tables and Figures

Table S2: Descriptive Statistics for clinical assessments, with mean, Standard Deviation (SD), and sample size (N), rounded to 1 digit.

Abbreviations: DSB = Digit Span Backward; DSF = Digit Span Forward; MRT = Mental Rotation Task; PEBL = Psychology experiment building language; TAP = Test of Attentional Performance; TMT = Trail-Making Test; WMS-LM =Wechsler Memory Scale "Logical Memory"

Assessment [Outcome measures]	N	Mean	SD
WMS-IV-LM 1 - free recall [total point score]	31	26.7	10.1
PEBL DSF [total point score]	26	5.9	2.2
PEBL DSB [total point score]	13	4.2	2.0
HOTAP [points x min^{-1}]	30	5.2	3.3
TAP "Go-NoGo" [median reaction time (ms)]	28	470.5	88.8
PEBL TMT-A [completion time (s)]	29	47.0	34.9
PEBL MRT [performance score]	18	43.0	10.8

Table S3: Descriptive Statistics for game metric data, with mean, Standard Deviation (SD), and sample size (N), rounded to 1 digit or 5 digits for values below 0.001

Game	Game metric	N	Mean	SD
Shopping Tour	Mean reaction time [ms]	31	6885.7	5305.1
	Mistakes [#]	31	1.4	0.9
	Collected items [#]	31	3.8	2.2
	Precision score [%]	31	76.1	15.7
Simon	Mean reaction time [ms]	26	1988.7	1753.1
	Point rate [ ]	26	0.00043	0.00023
Nomis	Mean reaction time [ms]	13	835.3	405.3
	Point rate [ ]	13	0.00046	0.00024
Targets	Hits [#]	30	9.1	3.6
	Misses [#]	30	1.5	2.1
	Point rate [ ]	30	0.00013	0.00006
Habitats	Mean reaction time [ms]	28	2021.3	278.7
	Point rate [ ]	28	0.00013	0.00007
Simple	Mean reaction time [ms]	29	1562.1	584.2
	Point rate [ ]	29	0.00011	0.00008
Gears	Mean reaction time [ms]	18	3840.2	1194.4
	Point rate [ ]	18	0.00010	0.00005
Tetris	Point score [ ]	10	48.7	64.0

Figures S1 - S8: Scatterplots of Correlation Analyses between exergame metrics with neuropsychological assessments.

r = Pearson's correlation coefficient; rs =Spearman's rank correlation coefficient.
for correlations meeting the H_A criteria: the red line represents the regression line, and the grey area the 95 % confidence interval.

Abbreviations: DSB = Digit Span Backward; DSF = Digit Span Forward; MRT = Mental Rotation Task; PEBL = Psychology experiment building language; TAP = Test of Attentional Performance; TMT = Trail-Making Test; WMS-LM =Wechsler Memory Scale "Logical Memory"

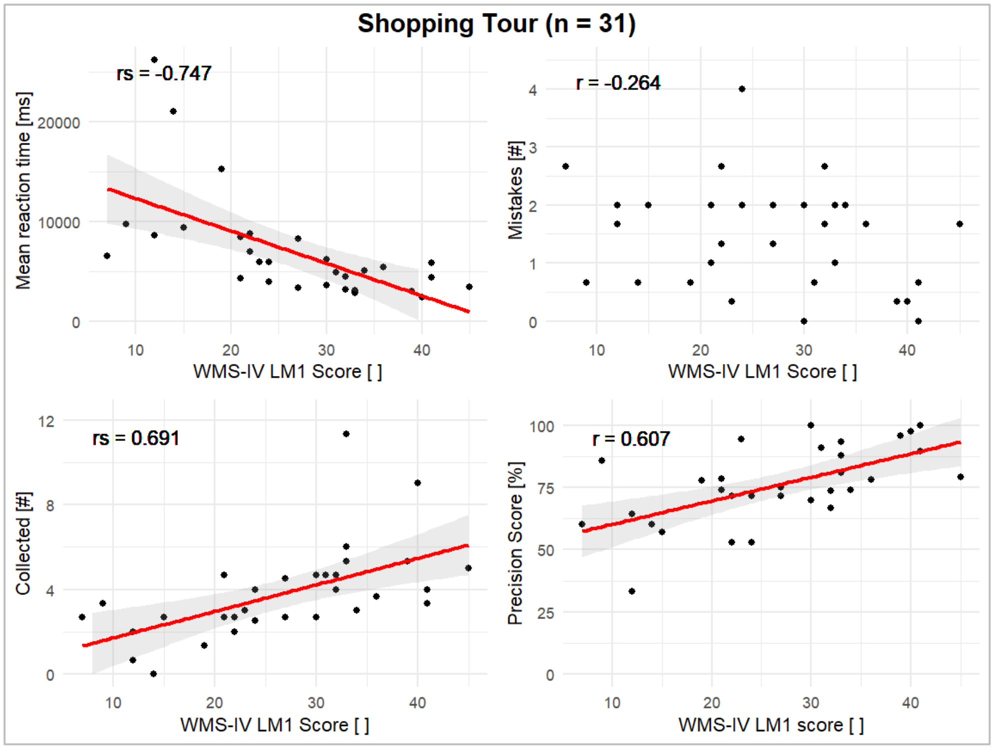

Figure S1

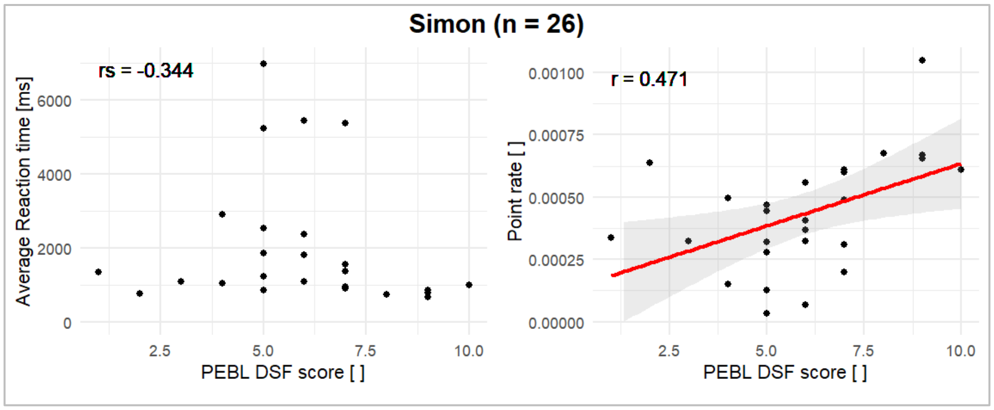

Figure S2

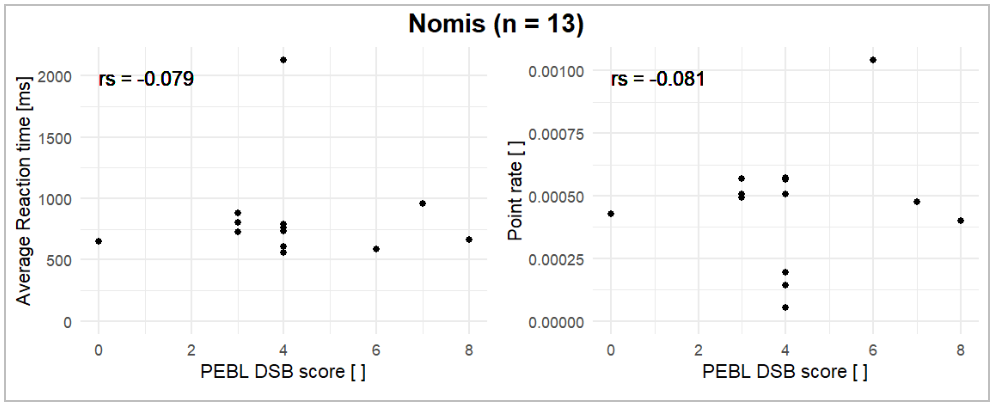

Figure S3

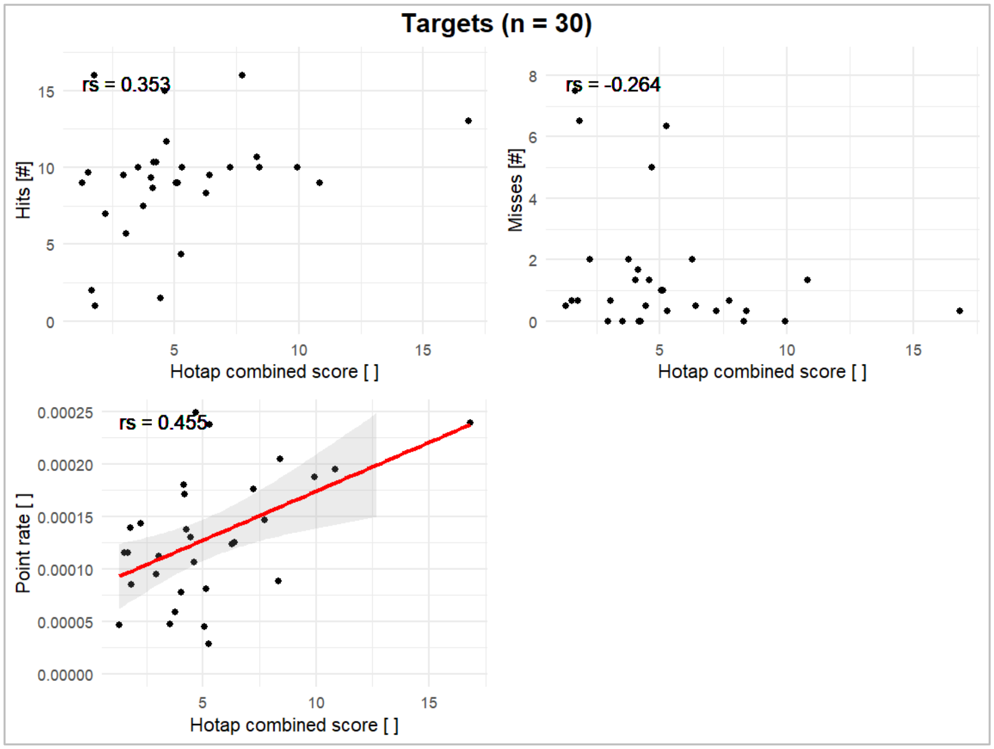

Figure S4

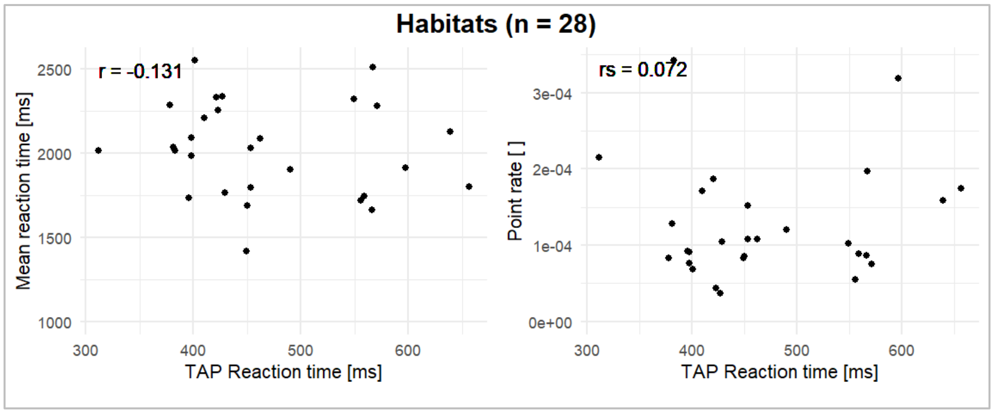

Figure S5

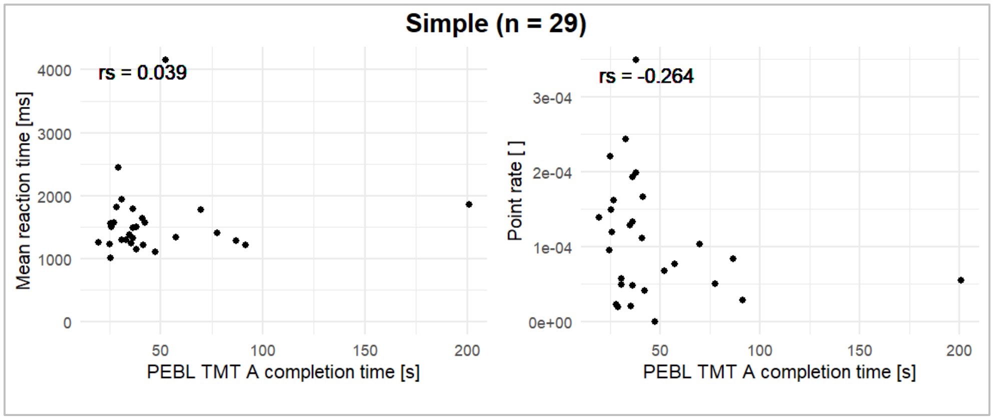

Figure S6

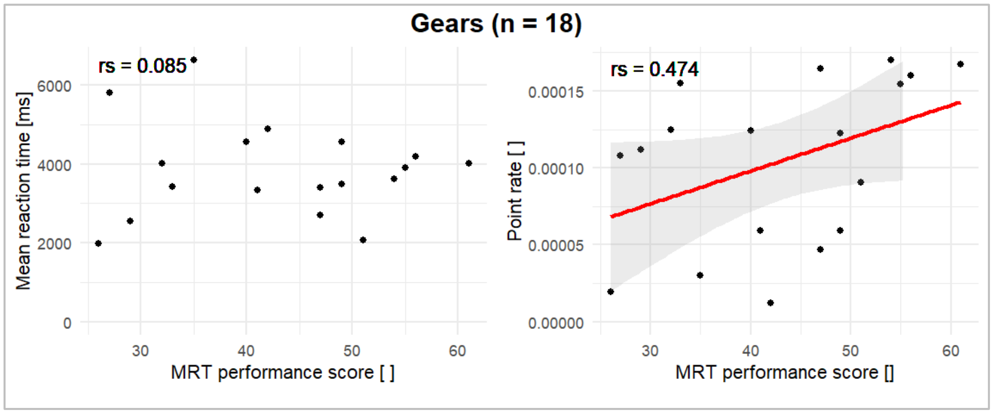

Figure S7

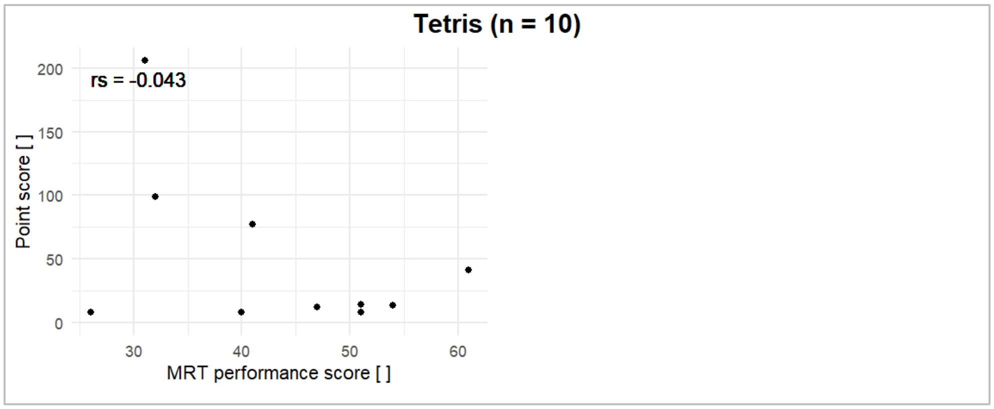
Tetris (n = 10)
rs = -0.043
Point score [ ]
MRT performance score [ ]
200
150
100
50
0
30
40
50
60

Figure S8

References

1. Manser P, et al. *Feasibility, usability, and acceptance of "Brain-IT"—A newly developed exergame-based training concept for the secondary prevention of mild neurocognitive disorder: a pilot randomized controlled trial*. Frontiers in Aging Neuroscience. 2023;15. doi: https://doi.org/10.3389/fnagi.2023.1163388.
2. Manser P, de Bruin ED. *"Brain-IT": Exergame training with biofeedback breathing in neurocognitive disorders*. Alzheimer's & dementia. 2024;20(7):4747-4764. doi: https://doi.org/https://doi.org/10.1002/alz.13913.
3. Manser P, de Bruin ED. *Making the Best Out of IT: Design and Development of Exergames for Older Adults With Mild Neurocognitive Disorder - A Methodological Paper*. Frontiers in Aging Neuroscience. 2021;13. doi: https://doi.org/10.3389/fnagi.2021.734012.
